# Estimation of the Antifungal Threshold of Thyme Essential Oil for Bread Preservation, Ensuring Consumer Acceptance and Product Quality

**DOI:** 10.3390/foods14203549

**Published:** 2025-10-18

**Authors:** Ricardo H. Hernández-Figueroa, Aurelio López-Malo, Nelly Ramírez-Corona, Emma Mani-López

**Affiliations:** Departamento de Ingeniería Química, Alimentos y Ambiental, Universidad de las Américas Puebla, Ex Hacienda Sta. Catarina Mártir, San Andrés Cholula, Puebla 72820, Mexico; nelly.ramirez@udlap.mx (N.R.-C.); emma.mani@udlap.mx (E.M.-L.)

**Keywords:** mold growth modelling, minimum inhibitory concentration, bread physical properties, thyme essential oil, bread sensory acceptability

## Abstract

The replacement of synthetic preservatives with natural alternatives is increasingly important in bakery production. This study examined the antifungal activity of thyme essential oil (TEO) against bread spoilage molds and its impact on product quality and consumer acceptance. TEO was tested at concentrations from 0 to 200 ppm against *Aspergillus flavus* and *Penicillium expansum* in bread and a model system, with mold responses modeled using the Gompertz equation. Because TEO affects the sensory qualities of bread, the kinetic parameters of mold growth were used to estimate the minimum inhibitory concentration (MIC), thereby ensuring a mold-free shelf life without significantly altering sensory properties. Bread samples were analyzed for pH, moisture, water activity, texture, specific volume, and sensory attributes (odor, flavor, texture, and acceptability). Residual thymol and carvacrol (measured using GC-MS) were also evaluated. The retention of thymol and carvacrol in baked bread was 75–80%. The tested TEO concentrations did not alter the moisture content, pH, or water activity of bread, while the specific volume was reduced and the width-to-height ratio increased as the TEO concentration increased. At concentrations below 100 ppm, TEO enhanced bread softness, while higher levels (>150 ppm) slightly increased hardness. Sensory testing showed no significant differences in color or texture (*p* > 0.05). At 50 ppm, TEO imparted a subtle thyme aroma and flavor, improving the sensory profile. At 100 and 150 ppm, the aroma and flavor became more pronounced and were well accepted. However, at 200 ppm, the thyme aroma and flavor decreased overall acceptance. In bread, the MIC of TEO for *A. flavus* ranges from 104.2 ppm (200 h delay) to 120.8 ppm (250 h), and for *P. expansum*, from 106.6 ppm (200 h) to 123.6 ppm (250 h). The MICs (100–125 ppm) fall within sensory acceptable scores, indicating that TEO can delay mold growth while maintaining bread quality. Moderate levels of TEO extended the mold-free shelf life of bread by providing microbial control and preserving its sensory properties.

## 1. Introduction

The focus on bread is not incidental, as it represents one of the most wasted staple foods worldwide, being susceptible to rapid spoilage [1]. Bread is the second-most-wasted food in the UK, with an annual wastage of 292,000 tons [1,2,3]. This high level of waste underscores the urgent need for improved preservation strategies to extend bread’s shelf life and reduce spoilage. The development of preservation methods that balance microbial safety, consumer acceptability, and sustainability goals is essential to mitigate both food loss and its associated environmental footprint.

Fungal spoilage, caused by *Aspergillus* and *Penicillium* species, is a major concern in bread preservation. Molds degrade quality, reduce shelf life, and pose safety risks by producing mycotoxins [4,5]. Mold spoilage significantly impacts bread making, decreasing shelf life and causing substantial waste. Common antifungal agents, such as calcium propionate and sorbic acid, effectively inhibit mold growth but are facing increasing scrutiny due to health concerns and rising consumer demand for products free from synthetic additives [5,6]. The growing consumer demand for clean-label products has driven the food industry to seek alternatives to synthetic preservatives [7,8]. As a result, the food industry is actively exploring natural options to meet these demands, such as essential oils (EOs).

Thyme (*Thymus vulgaris* L.) essential oil (TEO) has demonstrated significant antifungal activity attributed to compounds such as thymol and carvacrol [9]. Various studies have evaluated the TEO’s antifungal action against *Penicillium* and *Aspergillus* on bread, yielding variable findings. For instance, TEO (*Thymus zygis*) delayed mold growth (*Penicillium paneum*) on par-baked bread at concentrations of 0.2, 0.3, and 0.4 mL/100 g dough for 6.6, 10.1, and 13 days, respectively, at 22 °C, while moisture content and weight increased, and volume decreased [10]. Lower TEO concentrations (0.08, 0.15, and 0.20 mL/100 g dough) did not inhibit fungal growth (*P. paneum* and *Aspergillus niger*) on par-baked bread supplemented with sourdough (0, 15, and 30 g) during storage at 22 °C; even though TEO showed antifungal activity in similar conditions in vitro [11]. On bread, 50 μL/100 g flour reduced 54% *A. niger* and 64% *Penicillium* growth after 7 days stored at 25 °C [12]. Bread containing TEO (80 mg/100 g of flour) was reduced in volume and recorded color changes in the crumb and crust [13]. Volatile compounds from TEO have also shown antifungal action on dark rye bread for 7 days [14]; and for 14 days at 22 °C the growth of four *Aspergillus* (*A. flavus*, *A. parasiticus*, *A. ochraceus*, and *A. westerdijkiae*) was inhibited entirely when 500 μL/L_air_ was tested on bread [15]. Microencapsulated TEO exerted antifungal activity on cake (0.60 mg/mL, after 30 days at 25 °C) against contaminating molds (*Penicillium raistrickii* and *Aspergillus fumigatus*) [16]. In contrast, marginal inhibition of mold growth was achieved with an active film (made with a bacterial biopolymer added with 30% TEO), which delayed fungal growth on bread [17]. TEO’s antifungal activity is noticeable, and some insights into the mechanism of action against *A. flavus* have been revealed, showing that apoptosis occurs via nuclear condensation and plasma membrane damage [18].

Predictive mycology is primarily used to describe the inactivation, growth, or germination of fungi [19]. Kinetic models focus on fungal growth to understand the relationship between microorganisms and environmental factors and/or inhibitors. The time for visible growth of molds is more important than their growth kinetics because a food with a visible colony is considered spoiled [19]. Logistic and exponential statistical distributions can be applied to determine the time for visible growth, considering the variability within a population of spores [19]. Microbial stability is a key factor in bread preservation, where no mold growth is observed. Thus, the estimation of the lag phase of fungal growth (no growth) using mathematical models is important for determining the bread’s shelf life and could be more useful than kinetic approaches.

Effective mold growth control requires preservatives that are both potent and acceptable to consumers [5], ensuring both safety and quality in the food production chain. Although TEO is a great antifungal agent, the strong and unique odor associated with EO volatiles limits its use due to altering the typical smell/flavor of food. Safety and sensory quality are critical in staple foods, where consumer perception strongly influences purchasing decisions and overall food acceptance. As mentioned earlier, previous studies have focused on evaluating the antifungal activity of TEO (through microbial challenges) or on bread quality properties and visible mold growth during storage, using empirical amounts of TEO. In fungal growth modeling, both kinetic and probabilistic approaches have been employed to assess TEO activity in bread. Therefore, in this study, a comprehensive approach combining both methods was used to determine the inhibitory concentration of TEO needed to delay fungal growth, along with sensory scores assessing bread acceptability and quality features. Therefore, this study aims to evaluate the antimicrobial efficacy of TEO as a natural preservative against fungal contamination in model systems and traditional bread, specifically targeting *A. flavus* and *P. expansum*. Different TEO concentrations were tested, and the mold’s growth kinetics were fitted to experimental data. The minimum inhibitory concentration (MIC) of TEO was estimated using the model parameters and determined based on constraints related to the mold-free shelf-life target and minimal impact on sensory characteristics.

## 2. Materials and Methods

### 2.1. Essential Oil Extraction

EO was extracted from thyme (*Thymus vulgaris* L.) leaves purchased from Condimentos Naturales Tres Villas, Puebla, Mexico. Microwave-assisted hydro-distillation was conducted using a NEOS microwave extraction system (800 W, 60 Hz, NEOS, Milestone, Sorisole, Bergamo, Italy) with 200 g of thyme in distilled water at a 1:3 solid-to-water ratio [20]. The extraction process took 40 min at a stirring speed of 400 rpm [20]. The EO was weighed and analyzed following the methodology reported by Paris et al. [21] with slight modifications. Analyses were carried out using an Agilent 6850N gas chromatograph coupled to an Agilent 5975C mass spectrometer (Agilent Technologies, Santa Clara, CA, USA). Separation was performed on an HP-5 capillary column (30 m × 0.25 mm i.d., 0.25 μm film thickness). Helium was used as the carrier gas at a constant flow rate of 1.1 mL/min. The injector was maintained at 250 °C, operating in split mode, with an injection volume of 10 μL. The oven temperature was programmed as follows: initial temperature 60 °C (held for 2 min), increased at 10 °C/min to 250 °C, held for 10 min, and finally increased to 300 °C. The mass spectrometer was operated in electron ionization (EI) mode at 70 eV. The mass scan range was *m*/*z* 35–500. Compounds were identified by comparing their mass spectra with the NIST library and by calculating their retention indices (RI) relative to a homologous series of n-alkanes (C_8_–C_20_) analyzed under the same conditions.

### 2.2. Culture Preparation

*A. flavus* and *P. expansum* were obtained from the culture collection at the Food Microbiology Laboratory of the University of the Americas Puebla. Mold spores were harvested from 7-day-old potato dextrose agar (PDA, Bioxon, BD, Cuautitlán, Estado de Mexico, Mexico) slants using 5 mL of aqueous solution of 0.1% (*v*/*v*) Tween 80 (Sigma-Aldrich, St. Louis, MO, USA). The spore concentration in the suspension was determined using a Neubauer chamber, and the spore suspension was then diluted to achieve a concentration of ≈1 × 10^6^ spores/mL [22].

### 2.3. Antifungal Activity in Model Systems

The model systems were prepared using PDA (Bioxon, BD, Cuautitlán, Estado de Mexico, Mexico), adjusted to a pH of 5.6 using lactic acid solution (10%), and water activity of 0.96, adding sodium chloride. These values were selected to be similar to those of the formulated bread (bread a_w_ was determined in preliminary tests). This approach is commonly employed to assess the antimicrobial activity of EOs in culture media [23]. In this study, conditions were modified by adjusting PDA pH and water activity to match those of bread. The agar was sterilized, and the appropriate amount of TEO was added to achieve the desired concentrations of 0, 50, 100, 150, and 200 ppm. The formulated agar was then poured into sterilized Petri dishes (60 mm × 15 mm). The concentration range was based on prior studies reporting antifungal activity of TEO at similar levels, including sub-inhibitory and inhibitory doses. These concentrations also aimed to balance the antimicrobial effects with any potential sensory impact in bread formulations.

A 5 μL spore suspension was inoculated in the center of the agar and allowed to dry for 10 min under aseptic conditions. The inoculated plates were sealed with Parafilm, following incubation at 25 °C. Plates containing non-inoculated agar were used as controls. The water activity of the agar plates during the incubation period was controlled by placing them in sealed containers (avoiding anoxic conditions), with a plate on top and a water–glycerol solution (17.5% *w*/*w*) below that matched the a_w_ in the agar. The mold colony diameter was measured periodically with a digital caliper (Mitutoyo Corp., Kawasaki, Japan), taking measurements in two perpendicular directions. Three replicates were conducted for each mold and condition.

### 2.4. Bread Preparation

A standard direct dough process was employed using a basic formulation (wheat flour, 61%; water, 36%; salt, 1%; sugar, 1%; and yeast, 1%). A Legacy HL200 mixer/kneader (Hobart, Troy, OH, USA) was used to mix and knead the ingredients, and then incorporate TEO at concentrations of 0, 50, 100, 150, or 200 ppm into the dough, which was formed into 100-g pieces. The dough pieces were fermented for 60 min at 30 °C, then baked at 180 °C for 20 min in a Mini combo oven (Zucchelli Alpha, Trevenzuolo, Verona, Italy). After cooling at room temperature for 60 min, the bread was stored in sealed polyethylene bags at room temperature for analysis, not exceeding four hours [24].

### 2.5. Bread Inoculation

The methodology described by Dos Santos et al. [25] was used with some modifications. Bread pieces of each TEO concentration were sliced (thickness approximately 2.5 cm), cut into circles (approximately 6 g), and aseptically transferred to sterile 60 × 15 cm Petri dishes. The bread pieces were then compressed onto the dish’s surface with a plunger. 5 μL of the spore suspension was inoculated into the center of each bread sample. The plates were sealed with Parafilm, following incubation at 25 °C. Plates containing non-inoculated bread slices were used as controls. The water activity of the bread during the incubation period was controlled by placing the plates in sealed containers (avoiding anoxic conditions), with a plate on top and a water–glycerol solution (17.5% *w*/*w*) below that matched the bread a_w_. The inoculated plates were observed daily, and the colony diameter was measured using a digital caliper (Mitutoyo Corp., Kawasaki, Japan), taking measurements in two perpendicular directions. Three replicates were conducted for each mold and condition.

### 2.6. Mathematical Mold Growth Analysis

The modified Gompertz equation is often used to model mold growth because it effectively represents sigmoidal curves [26]. The growth curves of *P. expansum* and *A. flavus* were analyzed using Equation (1) with non-linear regression for parameter estimation.

(1) $$D=A\times \exp\left[-\exp\left[\left(\frac{\mu }{A}\right)\left(\lambda -t\right)+1\right]\right].$$

where *μ* is the maximum growth rate (mm/h), *A* is the maximum diameter observed, *λ* is the phase or lag time (h), and *D* is the colony diameter (mm) at time *t* (h).

To estimate the minimal inhibitory concentration of TEO, dose–response curves of the fungal response, specifically the growth rate and lag time at the tested concentrations, were fitted to empirical models. The logistic curve (Hill equation) and an exponential model were used. The logistic model can be applied in microbial growth studies, especially where inhibitory substances limit growth.

The model can be written as:

(2) $$D_{c\;}=\frac{D_{max}}{1+{\left[\frac{c}{IC_{50}}\right]}^{n}}.$$

where *D_c_* is the growth rate at TEO concentration (*c*), *D_max_* is the maximum rate in the absence of TEO, *IC*_50_ is the TEO concentration at which the growth rate is reduced by 50% (an indicator of inhibitory effectiveness), and *n* is the coefficient, representing the curve’s steepness.

An exponential model (Equation (3)) was used to analyze the observed lag times and estimate the MIC, selecting and defining the appropriate growth lag times.

(3) $$lag\;\left(h\right)=Aexp\left(-kC\right)\;$$

where *A* and *k* are the model parameters, and *C* is the TEO concentration in ppm.

### 2.7. Bread Analysis

Bread pH measurements were conducted using a pH meter model HI2210 (Hanna Instruments, Woonsocket, RI, USA) following the method AACC-02-52 [27]. The moisture content was determined using the AOAC 930.15 method [28], while the water activity (a_w_) analysis was performed using AquaLab equipment (Decagon Devices, Pullman, WA, USA). All measurements were conducted on whole bread, and the specific volume was determined following the AACC method 10-05 [27].

The width-to-height ratio was measured using a digital caliper. Bread hardness was assessed using a texture analyzer (EZ-SX, Shimadzu Corporation, Kyoto, Japan) on a 2.5 cm thick slice compressed to 50% using a stainless-steel cylinder probe (diameter = 25 mm) at a speed of 1 mm/s [29,30]. The maximum force (peak) was recorded as the force (in Newtons, N) from the curve analysis performed by the Trapezium X software (Shimadzu Corporation, Kyoto, Japan). All determinations were performed in triplicate.

Gas chromatography–mass spectrometry (GC-MS) was utilized to quantify residual thymol and carvacrol. A piece of bread (5 g) was mixed with 25 mL of ethyl acetate. The mixture was left to rest overnight, followed by 1 h of agitation. The ethyl acetate phase was recovered, filtered, and injected into a GC-MS for analysis, as described previously. TEO’s main components, thymol and carvacrol proportions, were quantified and compared with the proportions in the EO.

### 2.8. Sensory Analysis

Thirty untrained panelists evaluated the sensory quality of bread loaves based on odor, color, flavor, texture, and overall acceptability using a 9-point hedonic scale. Participants in the sensory evaluation tests provided informed consent by acknowledging the following statement: “I understand that my responses are confidential, and I consent to participate in this sensory evaluation.” Only those who agreed to the statement were eligible to participate and were informed that they could withdraw at any time without providing a reason. We explicitly stated, “The products being tested are safe for consumption.” We emphasized to participants that their data would remain confidential and only be shared with their explicit consent”.

### 2.9. Statistical Analysis

Each treatment was conducted in triplicate, and the results are shown as mean values with their standard deviations. The assumptions of normality and homogeneity of variance were checked before analysis, confirming that the data met the criteria for parametric testing. As a result, the data were statistically analyzed using Analysis of Variance (ANOVA). Pairwise comparisons of the mean values were performed with Tukey’s test at *p* < 0.05, using Minitab 20 statistical software (Minitab LLC, State College, PA, USA).

## 3. Results and Discussion

TEO’s extraction yield was 2% and its chemical composition is shown in Table 1. The primary identified compounds were thymol, p-cymene, carvacrol, caryophyllene, and γ-terpinene, which together represented over 88% of the total composition. Etri and Pluhár [31] and Pluhár et al. [32] noted that thymol concentration in TEO varies significantly due to chemotype, variety, region, and environmental conditions. Najar et al. [33] found that the ‘thymol’ chemotype contained thymol as its primary component, making up 49% to 51% of the EO, along with γ-terpinene and p-cymene. In contrast, the ‘linalool’ chemotype featured high levels of linalool (75%), with linalyl acetate (8.15%) and β-caryophyllene (3.2%) as additional main components. Antih et al. [34] analyzed three EOs from *Thymus vulgaris*. They found that thymol was the most abundant compound, with concentrations ranging from 29.52% to 48.65%. The second most prevalent was p-cymene, ranging from 12.75% to 25.35%, followed by carvacrol, with levels of 3.60% to 10.92%. At the same time, γ-terpinene was present in significant amounts (2.02% to 5.40%). The obtained and analyzed TEO (Table 1) contained trace amounts of borneol, terpinene-4-ol, α-pinene, linalool, and camphene, each making up approximately 1.0%.

### 3.1. Mold Growth Response

**Figure 1:**
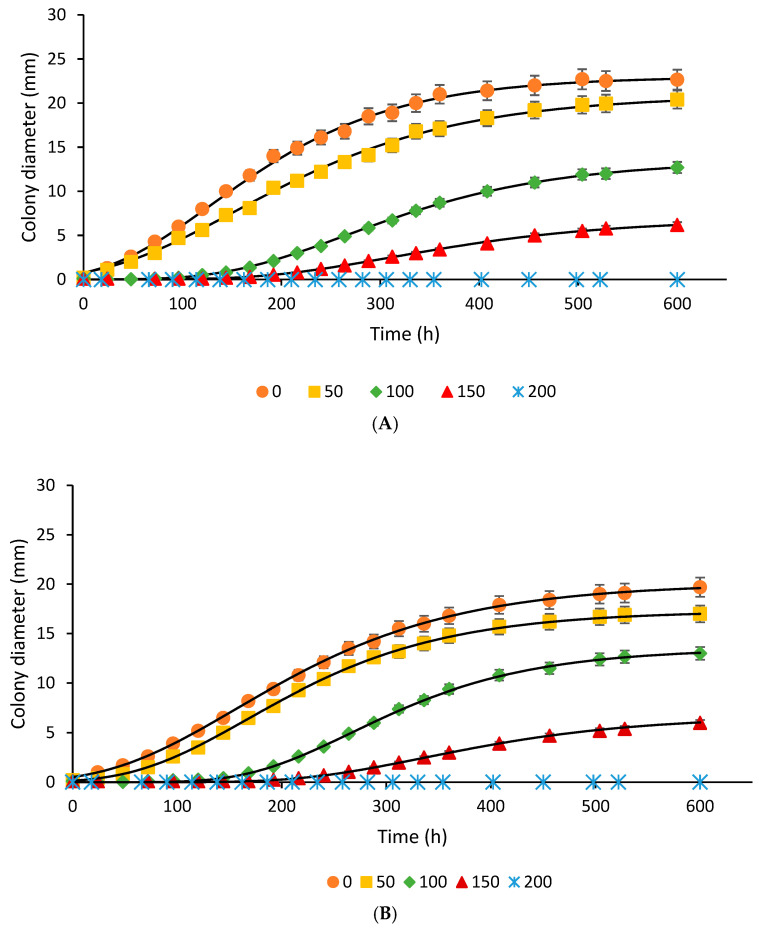
Effect of thyme essential oil concentration (● 0, ■ 50, ♦ 100, ▲ 150, and ∗ 200 ppm) on the growth of *Aspergillus flavus* in model systems (**A**) and bread (**B**). Symbols represent experimental data, and lines represent the Gompertz model fit.

**Figure 2:**
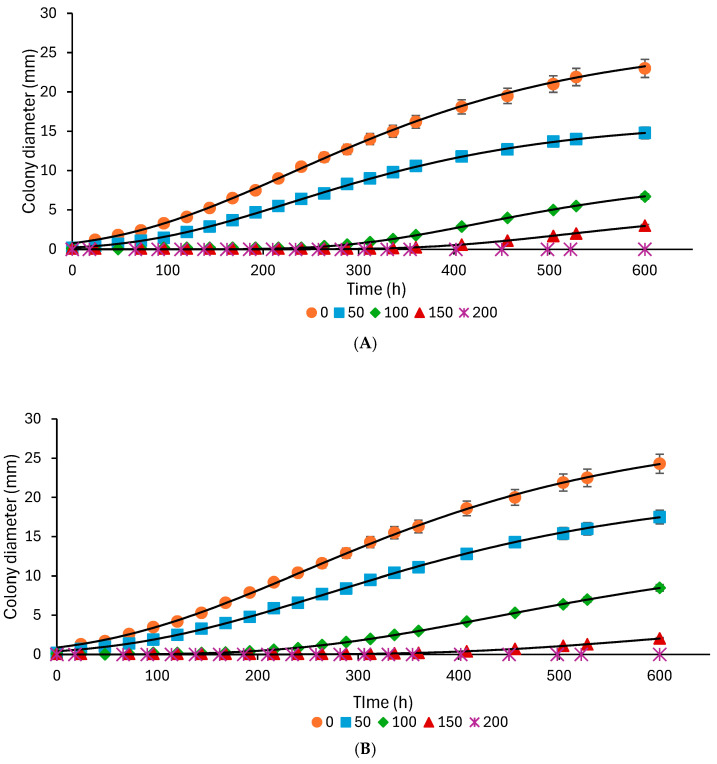
Effect of thyme essential oil concentration (● 0, ■ 50, ♦ 100, ▲ 150, and ∗ 200 ppm) on the growth of *Penicillium expansum* in model systems (**A**) and bread (**B**). Symbols represent experimental data, and lines represent the Gompertz model fit.

Figure 1A,B show the growth behavior of *A. flavus*, and Figure 2A,B display that of *P. expansum* during incubation in the model system and bread at different concentrations of TEO. In both the model system and bread, TEO effectively inhibits the growth of *A. flavus* and *P. expansum* in a concentration-dependent manner (Figure 1 and Figure 2). In the model system, *A. flavus* exhibits delayed germination and reduced maximum colony diameter with increasing TEO concentrations. At 200 ppm, fungal growth is significantly inhibited, with no visible growth observed for 620 h (approximately 25 days). Similarly, *P. expansum* exhibits prolonged lag times and reduced maximum growth at higher TEO concentrations, achieving complete inhibition after 620 h at 200 ppm. In bread, TEO demonstrates comparable antifungal effects. For *A. flavus*, TEO at 50–150 ppm delays germination and reduces maximum growth, while 200 ppm prevents visible mold for 25 days. *P. expansum* shows a similar response, with higher TEO concentrations lengthening lag times and decreasing colony size, finally inhibiting growth completely at 200 ppm. These findings highlight TEO’s potential as a natural antifungal agent for controlling mold growth in bread and food systems, as previously reported by Debonne et al. [11].

Research has shown that TEO exhibits a significant inhibitory action against various fungal species, including *Aspergillus* and *Penicillium*, common contaminants in bread [11,35]. The antifungal activity of TEO can be attributed to its main bioactive components (thymol and carvacrol), which are known for their potent antimicrobial properties. Increasing concentrations of TEO components increased the permeability of hyphal cell membranes, as indicated by increased extracellular conductivity, the release of cell constituents, and a decrease in extracellular pH, which are the primary mechanisms of action against molds. Thymol was particularly effective in inhibiting conidial production and hyphal growth, causing cell membrane damage through lipid peroxidation and interference with ergosterol biosynthesis [36]. These compounds disrupt fungal cell membranes and impair the production of ergosterol, a crucial component, leading to fungal cell death.

The modified Gompertz model was used to describe the behavior of *A. flavus* and *P. expansum* in response to TEO concentrations. This model is commonly applied in microbiology to describe microbial growth under the influence of various inhibitors, including antimicrobial agents.

**Figure 3:**
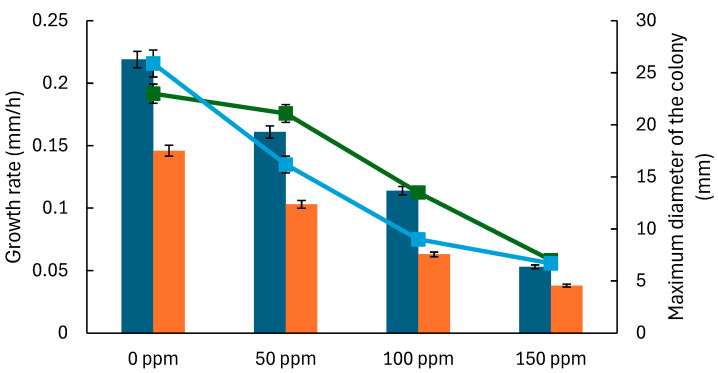
Parameters of the Gompertz model that describe the growth of *Aspergillus flavus* at various concentrations of thyme essential oil. Bars represent the growth rate, and lines represent the maximum colony diameter. Results are shown for model systems (blue bars, green lines) and bread (orange bars, blue lines).

**Figure 4:**
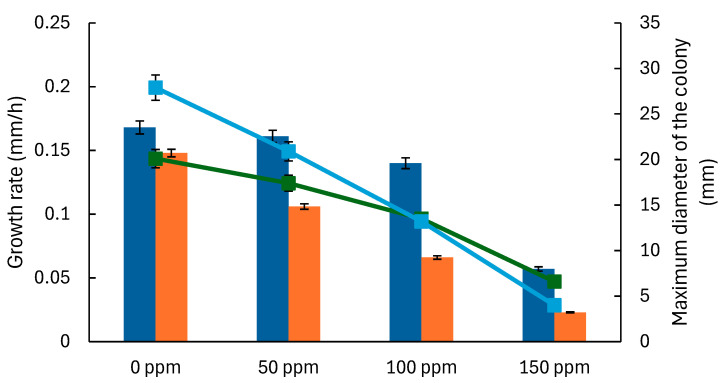
Parameters of the Gompertz model that describe the growth of *Penicillium expansum* at various concentrations of thyme essential oil. Bars represent the growth rate, and lines represent the maximum colony diameter. Results are shown for model systems (blue bars, green lines) and bread (orange bars, blue lines).

The model was used to analyze the impact of TEO concentration on mold growth by fitting the parameters for each system (model and bread) and concentration level. This enables the prediction of mold growth over time at various TEO concentrations and a comparison of growth dynamics between *A. flavus* and *P. expansum*. The model outputs help determine the effectiveness of TEO in inhibiting mold growth across different matrices, providing insights into its potential as a natural preservative in complex food systems, such as bread. Both molds display a minimum colony diameter and growth rate reduction as the TEO concentration increases, with complete inhibition observed at 200 ppm (Figure 3 and Figure 4). *A. flavus* appears slightly more sensitive to TEO, as evidenced by a sharper reduction in growth parameters at lower concentrations (50–100 ppm) in both the model system and bread (Figure 3).

**Figure 5:**
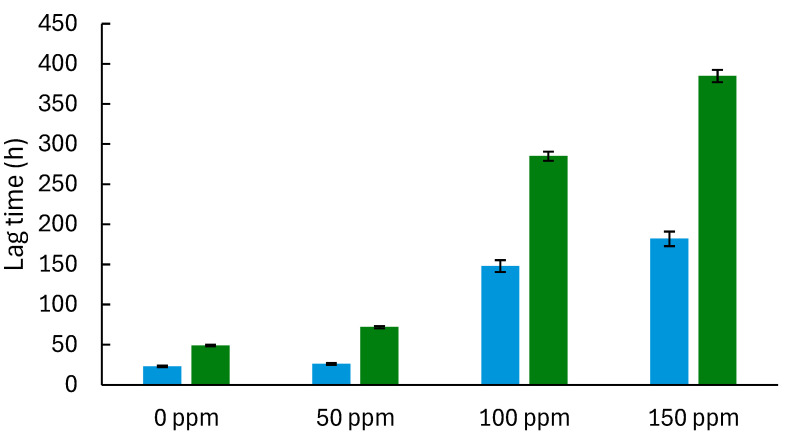
Gompertz model parameter (lag time) describing the growth of *Aspergillus flavus* at different concentrations of thyme essential oil in model systems (blue bars) and bread (green bars).

**Figure 6:**
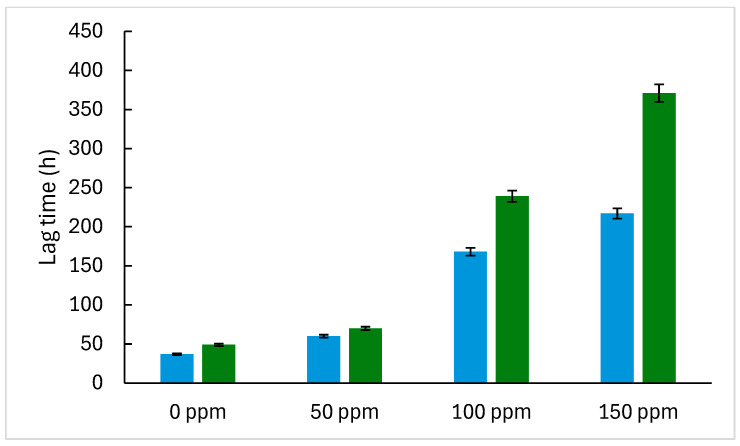
Gompertz model parameter (lag time) describing the growth of *Penicillium expansum* at different concentrations of thyme essential oil in model systems (blue bars) and bread (green bars).

The maximum colony diameter and growth rates for *A. flavus* and *P. expansum* are generally higher than those in the model system (Figure 3 and Figure 4), possibly due to the bread matrix’s more complex nutrient composition and structure, which can support more robust mold growth. In bread, the studied molds also show longer lag times (λ) at higher concentrations of TEO, indicating a delayed initiation of growth, likely due to the EO’s gradual diffusion and interactions with the bread matrix (Figure 5 and Figure 6). As TEO concentration increases, lag times (λ) increase for both molds, with more pronounced effects in bread (Figure 5 and Figure 6); at 100 ppm, *A. flavus* exhibits a 285 h lag phase and *P. expansum* a 239 h lag phase. This extended lag phase indicates a strong antimicrobial effect that delays spore germination and initial growth. The PDA model serves as a useful initial tool for evaluating the antifungal activity of EOs before testing them in actual food systems. Comparing both systems strengthens the conclusions by demonstrating how the antifungal effect is transferred from a model to a real food environment. Sriwattanachai et al. [37] found that TEO exhibited strong antifungal activity against *Penicillium* spp. The fungal radial growth was significantly inhibited (*p* < 0.05) in a dose-dependent manner using TEO, and the MIC (25 °C, pH 5, 5 days) were determined to be 50 ppm [38], resulting in complete inhibition of visible fungal growth. Using an in vitro assay with the broth microdilution method, the MIC of TEO for *P. expansum* was found to be 625 ppm [39].

### 3.2. Bread Physicochemical Properties

Table 2 presents the physicochemical properties of bread added with different TEO concentrations. Incorporating TEO did not affect bread’s moisture content, pH, and water activity values at the tested concentrations (*p* > 0.05). TEO impacts bread’s specific volume and width-to-height (W/H) ratio (Table 2). The W/H ratio measures the bread’s lateral spread in relation to its height after baking. This is influenced by the dough’s ability to hold gas and resist collapse. Bread with stronger gluten networks typically has lower W/H ratios. At low concentrations (50–100 ppm), the inhibitory compounds from TEO did not significantly delay yeast activity (*p* > 0.05), allowing adequate CO_2_ production and dough rise. At higher concentrations (150–200 ppm), the effects of thymol and carvacrol reduce gas production and dough expansion, resulting in greater lateral spread and an increase in the W/H ratio (*p* < 0.05). The dough retains its strength at 50–100 ppm, maintaining a balanced loaf shape with lower W/H ratios. Jurkaninová et al. [13] analyzed the effects of enriching wheat bread with 80 mg of TEO or lemongrass EO per 100 g of flour. They found that higher doses of EOs influenced the bread’s height and volume.

The final volume of a baked product is a key indicator of its quality. Specific volume (cm^3^/g) represents the volume of bread per unit of mass, and typical formulations without EOs yield specific volumes of 4.0–5.0 cm^3^/g [40]. The addition of TEO significantly reduces (*p* < 0.05) the specific volume of bread (Table 2). At low concentrations (50–100 ppm), the specific volume is comparable to the control (bread without TEO). However, higher concentrations (150–200 ppm) decreased the bread’s specific volume, indicating that the presence of TEO influences the overall structure and density of the bread product. Similarly, Debonne et al. [10] obtained reductions of volume (45–57%) in par-baked bread added with 0.3 or 0.4 mL TEO/100 g dough. In contrast, adding TEO or lemongrass EO to bread formulations typically does not significantly reduce volume compared to control [13]. EOs with strong antimicrobial properties may inhibit yeast, potentially decreasing bread volume due to reduced fermentation.

Hardness, namely firmness, was a commonly used index to describe bread quality, as changes in hardness typically reflect the loss of resilience. Control bread with normal gas retention and gluten structure shows moderate hardness (Table 2). At low TEO concentrations (50–100 ppm), minor effects on the gluten structure and moisture retention result in slightly increased softness, accompanied by a reduction in hardness. High TEO concentrations (150–200 ppm) result in denser bread with a slightly increased hardness, possibly attributed to the disruption of the gluten structure and reduced CO_2_ retention. Typical bread hardness ranges between 2 and 12 N, depending on the formulation and baking conditions. In textural analysis, no significant difference in firmness was observed between bread samples with and without the addition of ground thyme [41]. This indicates that appropriate additions of thyme would not cause any undesirable change in the hardness of bread.

### 3.3. TEO Components Concentration in Bread

Components such as thymol and carvacrol are thermally volatile, which can lead to potential loss during baking at 180 °C. Some compounds may also degrade into simpler substances at high temperatures. However, residual activity often persists, especially in the crumb, where heat penetration is lower. As expected, TEO losses were observed after bread baking. Reductions in the TEO content were detected, primarily attributed to the volatilization of thymol and carvacrol, as well as thermal degradation of these compounds. To estimate the recovery, the concentrations of thymol and carvacrol were quantified before and after baking, and the total post-baking content was expressed as a percentage of the initial content in TEO. The recovery ranged from 75% to 80%, indicating that although baking causes some compound losses, a significant portion of the active fraction remains in the bread matrix. The recovery of thymol plus carvacrol after baking was 80.0% at 50 ppm, 78.5% at 100 ppm, 76.9% at 150 ppm, and 75.3% at 200 ppm. These results demonstrate that, despite some loss, the remaining levels of thymol and carvacrol are sufficient to contribute to the antifungal activity observed in the bread samples. Debonne et al. [11] found that TEO retains its antifungal activity after autoclaving at 121 °C for 15 min, but they did not assess any potential inhibition compared to untreated EO. In contrast, Tomaino et al. [42] reported that the antifungal components remained unchanged after heat treatment at 180 °C for 10 min.

### 3.4. Sensory Analysis

When analyzing the sensory scores of the bread loaves, higher concentrations of TEO tend to generate more polarized responses due to the intensity of the thyme aroma and flavor. In contrast, lower concentrations are generally more acceptable (Table 3). This variation in preference is linked to TEO’s compounds responsible for the taste and aroma of primary thymol, as well as carvacrol, as the third component. Additionally, p-cymene and γ-terpinene are precursors to thymol, and linalool contributes with floral notes [43]. α-pinene provides pine-like scents. Other compounds, including terpinene-4-ol, borneol, caryophyllene, and camphene, which are present in smaller quantities, may also enhance the complexity of TEO.

The sensory analysis of bread with varying TEO concentrations revealed no significant (*p* > 0.05) changes in color and texture across the samples. The control bread (0 ppm) displayed typical characteristics and received good overall acceptance. At 50 ppm, TEO imparted a subtle and pleasant thyme aroma and flavor, enhancing the sensory profile without being overpowered. At 100 and 150 ppm, the thyme scent and flavor became more pronounced and were generally well-received, although some panelists found the flavor slightly strong. At 200 ppm, the intense thyme aroma and flavor diminished overall acceptance, as few panelists found them too strong.

In food science research, one of the primary challenges in creating bakery products enriched with EOs is finding a balance between protection and maintaining optimal sensory qualities [13]. Color, volume, crust crispiness, and crumb elasticity are key sensory attributes that influence consumer behavior and are closely connected to quality. Bota et al. [44] assessed the sensory characteristics of bread subjected to minimal concentrations of TEO vapors, demonstrating that such low levels do not adversely impact aroma or flavor and may occasionally be perceived favorably. Mani-López et al. [45] examined the effect of *P. expansum* inhibition on bread through the vapor-phase application of lemongrass EO, which demonstrated no significant difference in sensory acceptance at the MIC concentration. Bota et al. [44] analyzed the sensory qualities of bread samples made with TEO-fumigated wheat. The authors reported that bread from grain treated with 0.2% TEO received scores of 4.7/5.0 for overall acceptability and 4.8/5.0 for flavor, indicating high acceptability. For the same attributes, the bread sample made from grains treated with 0.4% TEO scored 4.6/5.0 for overall acceptability and 4.8/5.0 for flavor. These findings align with the fact that specific EO concentrations, adequate for inhibition, remain acceptable from a sensory perspective.

### 3.5. MIC Estimation Under Sensory Constraints for Mold-Free Bread

Several mathematical models can be used to estimate and compare the growth of microorganisms on various food matrices in response to different factors, including antimicrobial agents. This approach could significantly enhance the understanding of its preservative efficacy, being a helpful tool for estimating MIC when formulating model systems and food matrices with EOs as antimicrobial agents [46,47,48]. The MIC is defined as the lowest concentration of an antimicrobial agent that inhibits the growth of a microorganism after a specified incubation period [23]. One downside of recording this MIC value is that the established criterion relies on selecting the target value considered as inhibition. Furthermore, it has been pointed out that different incubation conditions, culture media, and numerous other factors influence the MIC, resulting in significant variance [49,50].

For molds, determining this value can be even more challenging, as viable cells are typically not counted during growth on surfaces (such as agar or food). Instead, mold’s radial growth rate and germination time (the period needed to develop a visible colony) are commonly utilized to assess the antimicrobial effectiveness of the selected agent. The techniques of agar dilution, also known as poisoning-agar or poisoning-food methods, are common methods for evaluating a compound’s efficacy and determining the MIC [51]. These methods involve choosing a medium favorable to mold growth, adding the target compound, inoculating it with the mold, and incubating for a designated time [23]. The mold’s mycelial growth is evaluated by measuring the diameter of its radial growth. By comparing this growth to that of a control (medium lacking the test compound), the efficacy of the compound can be determined [52], indicated by the percentage of mycelial growth inhibition relative to the fungal colony growth in the control [53]. By repeating this at various concentrations of the compound of interest and defining the inhibition criterion, the MIC is reported. This method is extensively employed in antifungal efficacy evaluations of EOs.

The Gompertz model parameters (Figure 3, Figure 4, Figure 5 and Figure 6) were fitted to Equations (2) and (3) to estimate MIC in terms of the growth rate and lag times. The obtained *D_max_*, *IC*_50_, and *n* values for each system (Table 4) suggest that growth inhibition varies among the model systems and bread, with TEO demonstrating different effectiveness levels across samples and molds. Regarding the lag times, the model (Equation (3)) adequately fits the experimental data (Table 5), with an average RMSE of 0.0018 and an R^2^ value greater than 0.970, allowing for a proper description of the dependence of this response on EO concentration.

As previously described, the reported MIC depends on the criterion chosen for the selected target value. Since this study evaluated the mold’s mycelial growth by measuring radial growth, the MIC was estimated using two approaches: (i) identifying the TEO concentration at which the growth rate is reduced, and (ii) determining the TEO concentration at which a lag is extended. Sensory analysis further constrained the practical application range of TEO. Bread with 50 ppm was described as having a pleasant thyme aroma that enhanced the flavor, while bread with 100–150 ppm produced more pronounced but still acceptable profiles. At 200 ppm, however, panelists reported an overpowering taste, resulting in significantly lower acceptance scores (*p* < 0.05). Therefore, MIC should be constrained to this acceptance range. As a reference, a lag longer than 8 days (194 h) would align with consumer requirements for preserving clean-label, mold-free bread.

For the first approach, a 75% reduction in growth rate was used to estimate the MIC for the molds, which were 143.2 ppm for *A. flavus* and 157.8 ppm for *P. expansum* in the model system. In contrast, these values were 134.3 ppm and 125.9 ppm, respectively, in bread. The second approach estimated the MIC that meets the lag criteria. Table 6 presents the estimated concentrations of TEO for selected delays in mold growth. The MIC values estimated from the two modeling approaches were comparable. The lag-based approach generated similar estimates (Table 6), with paired *t*-tests indicating no significant differences (*p* > 0.05) between MICs obtained from the two criteria: a 200–250 h lag extension and a 75% reduction in growth rate. This consistency highlights the robustness of predictive modeling for antifungal assessment (Table 6). In bread, *A. flavus* MIC of TEO ranges from 104.2 ppm (200 h growth delay) to 120.8 ppm for 250 h delay, and for *P. expansum*, it ranges from 106.6 ppm (200 h delay) to 123.6 ppm (250 h delay).

Notably, the estimated MICs (100–125 ppm) overlap with the sensory-acceptable range, indicating that TEO can effectively delay mold growth while maintaining bread quality. From an applied perspective, the extension of fungal lag times by ~200–250 h with concentrations of around 100–125 ppm in bread suggests a shelf-life increase of 8–10 days under typical storage conditions. Therefore, these TEO concentrations represent a practical strategy to extend bread shelf life while preserving consumer acceptance, supporting its potential as a natural antifungal solution for the baking industry.

Nieto [54] reviewed thyme as a natural antioxidant and antimicrobial agent in the food industry, aligning with consumer preferences for healthier options. While thyme can improve food stability, its use as a natural additive remains limited. Redondo-Blanco et al. [55] studied the antifungal effects of EOs like thyme on molds and their potential for food preservation. They noted that phytochemicals and EOs have antimicrobial properties that prevent food spoilage and mycotoxin contamination. The research highlighted three key plant nutraceuticals—terpenoids (thymol), polyphenols (resveratrol), and thiols (allicin)—which effectively inhibit fungal growth during mycelial development and germination. Maurya et al. [56] compiled EOs effectiveness against fungal pathogens such as *Aspergillus*, *Penicillium*, *Cladosporium*, and *Alternaria*. They reported that EOs can be considered viable alternatives to conventional preservatives due to their ability to enhance food shelf life. It was also shown to extend the shelf life of bread and cereal grains. In this study, TEO inhibited *A. flavus* and *P. expansum* at moderate levels while maintaining sensory acceptance.

To illustrate the potential impact of improved preservation, consider a medium-sized bakery producing 10,000 loaves of 800 g each per day, equivalent to approximately 8000 kg of bread daily. If the application of TEO-enabled fungal control were to extend the mold-free shelf life and reduce mold-related returns and discards by a conservative 15%, the avoided waste would amount to nearly 1200 kg per day. Over the course of a year, this translates to approximately 438 tons of bread that would otherwise have been discarded. The environmental significance of this reduction becomes clearer when considered in relation to bread’s carbon footprint. It should be noted that the production scale presented here is an illustrative example, based on values typical of medium-sized bakeries, and serves to contextualize the potential effect of TEO application on reducing food waste. Recent life-cycle assessments (LCAs) estimate the greenhouse gas emissions of bread to be approximately 0.8–1.2 kg CO_2_e per kilogram, depending on the type of bread and the production system. For example, emissions are reported at 0.81 kg CO_2_e/kg for Swedish rye bread, approximately 0.89 kg CO_2_e/kg for UK soft wheat bread, about 1.13 kg CO_2_e/kg for factory-gate white bread in Thailand, and between 0.98 and 1.24 kg CO_2_e per 800 g loaf in the UK [57,58,59,60,61,62,63]. Applying these values to the avoided waste scenario described above, the reduction could prevent between 350,400 and 525,600 kg CO_2_e annually [63,64,65]. This demonstrates that even modest improvements in shelf life can translate into significant sustainability benefits, simultaneously reducing food losses and lowering the environmental footprint of bread production.

Overall, replacing or reducing synthetic preservatives with a renewable, plant-based antifungal that maintains consumer acceptance supports sustainable food-processing goals, reduces unnecessary waste, and preserves product quality [66,67,68,69,70]. Bakeries can capitalize on market trends by developing innovative products that utilize the unique properties of EOs [13].

## 4. Conclusions

TEO exhibits significant antimicrobial activity against *A. flavus* and *P. expansum*, effectively prolonging the lag phase and reducing the growth rate of both molds as the concentration increases. While the EO’s inhibitory effect was consistent across 25 days in model systems, factors such as the bread’s components influenced outcomes, with higher maximum mold growth observed. At a concentration of 200 ppm, TEO effectively inhibited mold growth in bread, suggesting its potential as a natural antimicrobial agent. Sensory evaluations revealed that while low-to-medium concentrations (50–100 ppm) enhanced sensory acceptance, higher concentrations (200 ppm) polarized opinions due to the intensity of thyme’s flavor and aroma. The moisture content, pH, and water activity of the bread remained unchanged at the tested TEO concentrations, while increasing TEO decreased the specific volume and the width-to-height ratio increased. Additionally, bread hardness increased with higher TEO levels. The thymol and carvacrol components of TEO remained at levels between 75% and 80% after baking, which was sufficient to provide antifungal activity in the bread. Considering the delay in mold growth and the sensory quality of the bread, the recommended TEO concentration for bread preservation was estimated to be around 100–125 ppm. This study demonstrates TEO’s potential as a natural preservative, supporting the development of bakery products that achieve a balance between microbial control and sensory acceptance. This meets the growing demand for “clean-label” solutions and aligns with sustainable food-processing goals by reducing avoidable waste.

## Figures and Tables

**Table 1:** Major components of thyme (*Thymus vulgaris*) essential oil.

Compound	Proportion (%)	Retention Index
Thymol	33.25 ± 0.67	1290
p-Cymene	30.5 ± 0.76	1025
Carvacrol	12.5 ± 0.38	1298
Caryophyllene	6.5 ± 0.13	1419
γ-Terpinene	5.5 ± 0.14	1057
Borneol	4.0 ± 0.12	1165
Terpinene-4-ol	1.75 ± 0.04	1177
α-Pinene	1.55 ± 0.04	939
Linalool	1.4 ± 0.04	1096
Camphene	1.25 ± 0.03	954

**Table 2:** Bread’s physicochemical and quality properties.

TEO Concentration (ppm)	Moisture Content (%)	Water Activity	pH	W/H Ratio	Specific Volume (cm^3^/g)	Hardness (N)
0	40.2 ± 0.4 ^a^	0.955 ± 0.005 ^a^	5.65 ± 0.03 ^a^	0.98 ± 0.06 ^a,b^	5.01 ± 0.14 ^a^	4.25 ± 0.25 ^b,c^
50	41.0 ± 0.5 ^a^	0.960 ± 0.007 ^a^	5.61 ± 0.07 ^a^	0.97 ± 0.06 ^b^	4.99 ± 0.12 ^a^	3.85 ± 0.15 ^c^
100	40.1 ± 0.3 ^a^	0.945 ± 0.004 ^a^	5.67 ± 0.06 ^a^	1.00 ± 0.05 ^a,b^	4.74 ± 0.16 ^a,b^	3.98 ± 0.18 ^c^
150	40.8 ± 0.6 ^a^	0.950 ± 0.003 ^a^	5.66 ± 0.03 ^a^	1.05 ± 0.04 ^a,b^	4.46 ± 0.15 ^b^	4.50 ± 0.20 ^a,b^
200	40.5 ± 0.4 ^a^	0.957 ± 0.008 ^a^	5.63 ± 0.04 ^a^	1.10 ± 0.05 ^a^	4.38 ± 0.20 ^b^	4.80 ± 0.11 ^a^

In each column, means that do not share a superscript letter are significantly different (*p* < 0.05).

**Table 3:** Scores of the sensory evaluation of the loaves of bread added with thyme essential oil (TEO).

TEO Concentration (ppm)	Color	Odor	Texture	Flavor	Overall Acceptability
0	7.50 ± 0.80 ^a^	7.00 ± 0.60 ^a^	7.50 ± 0.40 ^a^	7.20 ± 0.40 ^a^	7.50 ± 0.40 ^a^
50	7.50 ± 0.75 ^a^	7.40 ± 0.80 ^a^	7.50 ± 0.50 ^a^	7.50 ± 0.40 ^a^	7.70 ± 0.50 ^a^
100	7.40 ± 0.85 ^a^	7.20 ± 0.90 ^a^	7.50 ± 0.60 ^a^	7.10 ± 0.45 ^a^	7.30 ± 0.60 ^a,b^
150	7.30 ± 0.90 ^a^	7.00 ± 0.80 ^a^	7.40 ± 0.50 ^a^	6.80 ± 0.50 ^a,b^	6.90 ± 0.55 ^a,b^
200	7.40 ± 0.95 ^a^	6.80 ± 0.90 ^a^	7.50 ± 0.70 ^a^	6.10 ± 0.60 ^b^	6.10 ± 0.75 ^b^

In each column, means that do not share a superscript letter are significantly different (*p* < 0.05).

**Table 4:** Dose–response model parameters (Equation (2)) describing the effect of thyme essential oil concentration on mold growth rate.

Parameter	*Aspergillus flavus*		*Penicillium expansum*	
	Model System	Bread	Model System	Bread
*IC*_50_	94.5	82.6	134.1	82.6
*N*	2.64	2.26	6.76	2.61
*D_max_*	0.211	0.144	0.164	0.145
RMSE	0.0163	0.0095	0.0059	0.0087
R^2^	0.979	0.984	0.997	0.988

**Table 5:** Exponential model parameters (Equation (3)) describing the effect of thyme essential oil concentration on mold lag time.

Parameter	*Aspergillus flavus*		*Penicillium expansum*	
	Model system	Bread	Model system	Bread
*A*	22.55	49.53	37.65	49.51
*K*	0.0156	0.0134	0.0133	0.0131

**Table 6:** Estimated thyme essential oil minimal inhibitory concentrations (ppm) to delay growth or reduce the growth rate of *Aspergillus flavus* and *Penicillium expansum* in papa dextrose agar (PDA) and bread.

Mold	System Tested	Lag Time (h)			Growth Rate Reduction
		150	200	250	75%
*A. flavus*	PDA	121.5	139.9	154.2	143.2
*P. expansum*	PDA	103.9	125.6	142.3	157.8
*A. flavus*	Bread	82.7	104.2	120.8	134.3
*P. expansum*	Bread	84.6	106.6	123.6	125.9

## Data Availability

The original contributions presented in this study are included in the article. Further inquiries can be directed to the corresponding authors.
